# Boosting agricultural green development: Does socialized service matter?

**DOI:** 10.1371/journal.pone.0306055

**Published:** 2024-06-24

**Authors:** Yongqi Yu, Zexin Chi, Yanfeng Yu, Junjie Zhao, Liulin Peng

**Affiliations:** 1 School of Economics and Management, Jiangxi Agricultural University, Nanchang, China; 2 Institute of Agricultural Economics and Information, Jiangxi Academy of Agricultural Sciences, Nanchang, China; 3 Institute of Agricultural Economics and Development, Chinese Academy of Agricultural Sciences, Beijing, China; Zhongnan University of Economics and Law, CHINA

## Abstract

Agricultural socialized service is gradually emerging as a new stimulus for enhancing the agricultural production environment. However, their precise impact on improving the agricultural ecological environment and promoting the green development of agriculture remains incompletely understood. Therefore, leveraging panel data spanning from 2003 to 2020 across 31 provinces in China, this study utilizes the bidirectional fixed effect model, moderating effect model, and spatial Durbin model to systematically assess the influence of agricultural socialized services on agricultural green development and its spatial ramifications. The findings show that (I) agricultural socialized services significantly contribute to promoting agricultural green development, particularly in regions with lower aging demographics. (II) The application of the spatial Durbin model reveals that this promotional effect does not exhibit significant spatial spillover effect. (III) The role of agricultural socialized services in fostering agricultural green development can be significantly enhanced by advancements in land transfer, agricultural technological innovations, and the improvement of rural human capital. In conclusion, the study provides a set of policy recommendations that include government financial support, facilitating land transfer, improving rural education and technical training, and promoting green production technologies to effectively promote agricultural green development.

## Introduction

Since 2004, China has achieved the remarkable milestone of nineteen consecutive years of successful grain production harvests. However, it is also confronted with challenges such as a decline in cultivated land productivity [1,2], excessive use of pesticides and fertilizers [3,4], tightening resource constraints [5], severe environmental pollution [6], and ecosystem degradation [7]. These issues pose a serious threat to China’s food security [8] and sustainable agricultural development [9,10], urging the agricultural sector to urgently pursue intensive and green growth models [11]. In this context, the rapid development of agricultural socialized services seems to provide an opportunity to promote the green transformation of agricultural production.

As the primary method to foster an organic connection between small farmers and modern agriculture [12], agricultural socialized services not only alleviate the issue of insufficient agricultural production power and enhance agricultural productivity [13], but also emerge as the strategic focal point for promoting agricultural modernization and the primary driving force for moderately scaled agricultural management [14]. It is considered the third driving force in the history of China’s agricultural modernization by the academic community [15]. In recent years, the output value of China’s agricultural productive service industry has achieved rapid growth, and the number of service organizations and service areas has also expanded, providing strong support for the green transformation of agriculture. According to data from China’s Ministry of Agriculture and Rural Affairs in 2021, the output value of China’s agricultural productive service industry had reached 774.77 billion yuan, representing a 7.56-fold increase from 90.53 billion yuan in 2003. Various socialized service organizations nationwide numbered 1.04 million, serving an area exceeding 125 million hectares and catering to over 89 million small farmers. Practices have proven that agricultural socialized services play an important role in promoting green agricultural production. It can assist small farmers in adopting advanced agricultural green production technology [16], optimizing agricultural production input factors [17,18], transforming traditional agricultural models [19], and guiding farmers to enhance the protection of cultivated land quality [20]. Given these comprehensive benefits, it is evident that agricultural socialized services are swiftly emerging as a crucial factor in catalyzing the transformation of China’s agricultural green development throughout its entire process.

## Literature review

At present, agricultural socialized services and agricultural green development (AGD) have become the research hotspots in the policy field of agriculture, rural areas, and farmers. Through an in-depth review of the existing literature, we find that the academic community has not reached a consensus on whether agricultural socialized services promote the green development of agriculture. Although the mainstream view tends to affirm the positive role of agricultural socialized services in AGD, there are also some opposing views that cannot be ignored. From a positive perspective, agricultural socialized services play an important role in promoting AGD. For example, several studies have demonstrated that agricultural socialized services can reduce agricultural production costs, increase farmers’ willingness to engage in green production [21], and encourage them to adopt green production technologies as well as water and soil conservation measures [5,22–25]. In addition, under the technical guidance of these services, farmers can be encouraged to master the appropriate application of pesticides and fertilizers [26,27], thereby enhancing the overall green total factor productivity of agriculture [22,28–30]. However, from the perspective of research on the relationship between agricultural socialized services and agricultural production efficiency, some scholars also argue that agricultural socialized services hinder agricultural production efficiency. For instance, studies have shown that the provision of effective services in pest control may have a negative impact on the efficiency of rice production technology [31]. At the same time, some public agricultural extension services may lead to increased fertilizer application and total expenditure [18,32]. In addition, some one-time production technology training has also failed to effectively reduce the amount of nitrogen fertilizer applied to farmers [4]. Similarly, Bambio and Agha (2018) also found that the involvement of agricultural socialized service organizations has exacerbated the issue of excessive fertilization [33].

Furthermore, most scholars have extensively explored the research on the mechanism of influence of agricultural socialized services on AGD from a micro perspective. Specifically, researchers have investigated the influence mechanism of agricultural socialized services at various stages (e.g., pre-production, mid-production, and post-production) on farmers’ environmentally friendly production behavior. This includes reducing the use of pesticides and chemical fertilizers, as well as their willingness to embrace green agricultural technologies [22,34–36]. Meanwhile, the role of factors such as scale of operation, land fragmentation, land size, specialized division of labor, farm size, and technology adoption in the process of agricultural socialized services affecting AGD has also been extended to study [17,22,37–40].

The existing research in related fields has indeed yielded impressive results, yet several crucial aspects remain unexplored. Firstly, regional differences in the impact of agricultural socialized services on AGD are not sufficiently explored. Particularly, there is limited literature that delves deeply into the spatial effects of agricultural socialized services on AGD. With the continuous improvement of the agricultural socialized services system, the operation of cross-regional socialized services is deepening. In light of the variations in resource endowment and the level of AGD among different regions, it is essential for us to investigate the regional disparities and spatial spillover effect of the impact of agricultural socialized services on AGD. Secondly, existing studies have mostly focused on the micro level to explore how agricultural socialized services affect farmers’ green production behaviors. However, research on the impact of agricultural social services on AGD and its mechanisms of action remains limited at the macro level, such as the regional or national level. It is therefore necessary to move further to the macro level and seek a more comprehensive understanding of the important role of agricultural socialized services in promoting AGD. Moreover, considering the accelerating pace of land transfer in China, along with the rising educational level of farmers and the widespread adoption of advanced agricultural production technologies, it is crucial to examine the role of land transfer, rural human capital, and agricultural technological innovation in influencing the impact of agricultural socialized services on AGD. This will provide us with deeper and more comprehensive insights that can drive the advancement of AGD.

Based on existing research, this paper expands and delves deeper into the subject matter. The marginal contributions of this study primarily reside in two aspects. Firstly, this paper fully considers the spatial effect of the influence of agricultural socialized services on AGD. Most of the previous studies discussed the relationship between agricultural socialized services and AGD from a single perspective, and paid less attention to the spatial effect of agricultural socialized services. Therefore, by using the method of spatial econometrics, this paper makes a more comprehensive analysis of the spatial impact of agricultural socialized services on AGD, so as to enrich the research content and improve the practicability of the research. Secondly, this paper re-verifies the influence of agricultural socialized services on AGD from a macro perspective. This revalidation process not only helps to consolidate the findings of existing studies, but also provides policymakers with a more comprehensive and in-depth reference basis. Thirdly, we further explore the role of land transfer, rural human capital and agricultural technological innovation in the impact of agricultural socialized services on AGD on the basis of existing research results. On the one hand, this helps to further clarify the mechanism of agricultural socialized services on AGD, and on the other hand, it also provides more targeted guidance for policy makers.

The remainder of this paper is structured as follows: The Theoretical Analysis section explains the theoretical mechanism and proposes research hypotheses., The Methods section introduces variables, data sources, and analytical models. The Discussion of Results section offers empirical research findings and their analysis, and the Conclusion, Policy Implications and Limitations section summarizes the conclusions and puts forward policy recommendations, as well as the shortcomings and future intentions of this study.

## Theoretical analysis

### Impact of agricultural socialized services on AGD and its spatial effects

Yang et al. [18], Zhang et al. [39], Ma et al. [41], and Li et al. [42] found that agricultural socialized services can promote the adoption of agricultural green production technologies, improve the efficiency of agricultural production, and contribute to AGD. Specifically, on the one hand, the professional division of labor formed by agricultural socialized services improve the efficiency of "mechanical substitution for labor," making farmers more inclined to adopt cost-effective production methods and delegate high-cost production aspects of agriculture to more efficient agricultural social service organizations. This leads to higher environmental and economic benefits [43]. On the other hand, as the number of links through which farmers adopt agricultural socialized services increases, the factor substitution effect of these services expands, alleviating the factor constraint of labor shortage in farmers’ households and providing a practical pathway for the introduction of human and knowledge capital [35]. Moreover, as factor substitution deepens, the likelihood of farmers participating in agricultural green production will undoubtedly increase. This, in turn, will encourage more farmers to engage in the green production cycle. In addition, socialized services can also encourage farmers to use reduced inputs and adopt green production technologies through the technology improvement effect. This, in turn, enhances the efficiency of green agricultural production and facilitates the transformation of AGD. Therefore, hypothesis H1a is proposed in this paper.

**H1a.** Agricultural socialized services can positively promote AGD.

Agricultural socialized services, as an important production factor characterized by intensive knowledge and technology, strong mobility, and high integration [44,45], can leverage its unique features to overcome spatial limitations. It not only influences the agricultural green development of the region but also disseminates advanced knowledge, green technology, and human capital to neighboring and even non-neighboring regions through inter-regional agricultural operation services [46,47] and information transfer [48]. This process drives these regions towards intensive and large-scale production, subsequently impacting their AGD. Therefore, hypothesis H1b is proposed in this paper.

**H1b.** The impact of agricultural socialized services on AGD has a positive spatial spillover effect.

### Moderating effects of land transfer, technological innovation and rural human capital

Currently, in the process of promoting the development of Chinese-style agricultural modernization, China has introduced a series of policies to facilitate rural land transfer, advance basic agricultural science research, and enhance the quality and skill level of farmers. These initiatives have transformed the operational landscape for agricultural development. So, what is the role of land transfer, technological innovation, and rural human capital in the process of agricultural socialized services affecting AGD, and do they have moderating effects? It is worth investigating these issues.

#### Agricultural socialized services, land transfer and AGD

For a long time, China’s national condition of "big country and small farmers" has determined the small-scale operation of farmers. This situation has led to the fragmentation of rural land and the use of crude production methods, which in turn has constrained AGD in terms of mechanization and specialization due to land fragmentation [39]. Land transfer is an important approach to tackle the issue of land fragmentation and achieve AGD [49]. It can not only promote the transfer of land resources from low-productivity farmers to high-productivity farmers [50], which can optimize the allocation of land resources [51], but also provide the scale of land needed for agricultural socialized services. This can reduce the cost of agricultural operation and promote the agglomeration of agricultural socialized services and the development of socialized service organizations [52]. In addition, the "scale effect of agricultural land" formed by land transfer can significantly promote the reduction of agricultural inputs such as pesticides and fertilizers [53]. The resulting "green benefits" make farmers more inclined to use agricultural socialized services for the purpose of green production, thus improving the efficiency of green agricultural production. Therefore, hypothesis H2 is proposed in this paper.

**H2.** Land transfer positively regulates the impact of agricultural socialized services on AGD.

#### Agricultural socialized services, agricultural technology innovation and AGD

Technological innovation is an essential driving force for economic growth and a critical factor in promoting green development in agriculture [54–56]. With the innovation and popularization of agricultural technologies such as cultivated land technology, agricultural pollution control technology, and machinery technology, the forms of agricultural socialized services are diversifying, and the scope of services is expanding. It is no longer limited to specific operations such as agricultural machinery, plant protection, and seedling breeding. It will also disseminate advanced production technologies, methods, and concepts to farmers through knowledge spillovers. This will encourage them to change their previous production practices, thereby promoting green agricultural production. Meanwhile, the economic impact of agricultural technology innovation will encourage farmers to engage more actively in various agricultural socialized services and opt for green production technologies for their own benefit. This will further enhance the green effect of agricultural socialized services. Therefore, if more farmers can adopt green production technologies such as no-till technology, biological control technology, and large farm machinery through agricultural socialized services, they can greatly reduce the use of agricultural chemicals and improve the efficiency of energy and farm machinery use. This, in turn, will lead to the sustainable development of agriculture in the entire region. Therefore, hypothesis H3 is proposed in this paper.

H3. Agricultural technology innovation positively regulates the impact of agricultural socialized services on AGD.

#### Agricultural socialized services, rural human capital and AGD

Labor is an essential input factor for agricultural production. From the labor perspective, the quality of human capital influences the adoption of agricultural socialized services by farm households, subsequently impacting the level of AGD. Educational attainment is a crucial indicator of human capital. Assuming that farmers have a low level of education, they may reject new technologies, skills, and organizational models due to their traditional beliefs and limited capacity for learning [57]. This resistance hinders the development of agricultural socialized service organizations and impedes the adoption of green technologies and AGD. As the level of education increases, farmers are more likely to utilize available resources effectively for agricultural production [58]. As people gain a better understanding of the economic and environmental benefits of socialized agricultural services, they are more likely to adopt them [59]. At present, China’s rural population is aging, but the overall quality of human capital is slowly improving. According to the statistics from the China Human Capital Report 2022, the average education level of the rural workforce has increased from 7.5 years in 2001 to 9.2 years in 2020. This improvement provides favorable conditions for the ecological impact of agricultural socialized services. Therefore, hypothesis H4 is proposed in this paper.

**H4.** Rural human capital positively regulates the impact of socialized agricultural services on AGD.

**Fig 1:**
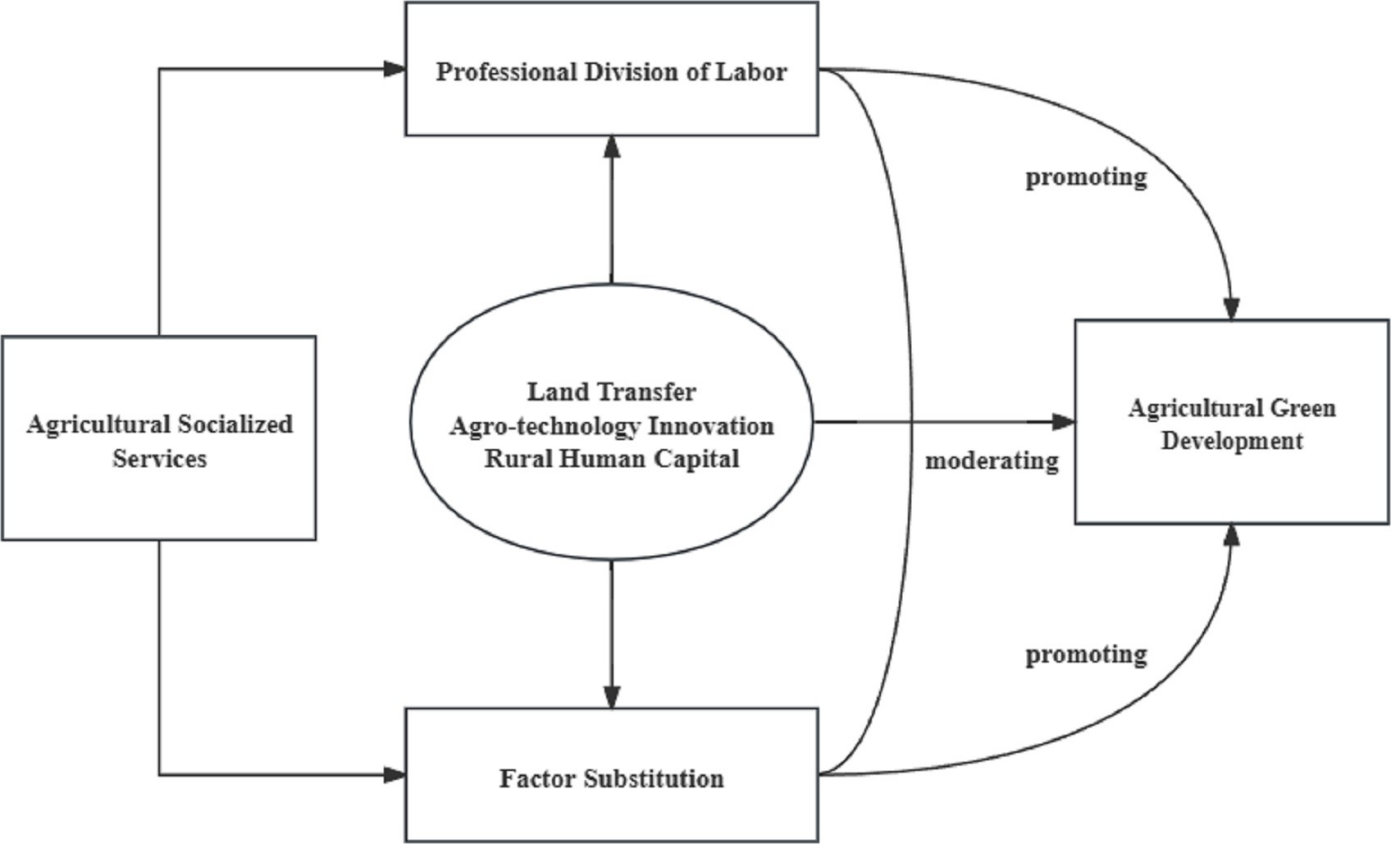
Theoretical research framework.

Combined with the theoretical analysis above, this study systematically describes the relationship between agricultural socialized services and AGD. It also examines the moderating effects of land transfer, agricultural technology innovation and rural human capital on the impact of agricultural socialized services on AGD. The theoretical framework is derived by combining related studies, as illustrated in Fig 1.

## Methods

### Variables description

(1) Dependent variable. AGD is a comprehensive concept, the core meaning of which is to coordinate the economic, social, environmental, and ecological benefits of agricultural development to achieve the harmonization of the four effects[60].At present, many scholars estimate green total factor productivity from the perspective of input-output [27,61–64], this provides a good method for measuring the level of AGD, but it cannot completely cover the four aspects of economy, society, environment and ecology. This paper aims to develop a system of indicators that cover these four aspects to measure the level of AGD. Therefore, based on the availability and applicability of data, and following the methodology of Wei et al. [65] and Guo and Xu [66], 19 indicators have been chosen from four perspectives: resource conservation, environmental friendliness, ecological conservation, and economic benefits to establish an indicator system for assessing the level of AGD. In this paper, the entropy weight method is used to calculate the level of green development in agriculture, and the corresponding indicators and weights are presented in Table 1.

**Table 1:** AGD level index system.

Dimensions	Components	Indicators	Unit	Attribute	Weights		
					W_en	W_cri	W_con
Resources Conservation	Agricultural water efficiency	Agricultural water consumption / total agricultural output	m^3^ / 10000 yuan	-	0.006	0.066	0.036
	Reproduction index	Crop sown area/year-end arable land*100%	%	-	0.021	0.047	0.034
	Total power of agricultural machinery per unit sown area	Total agricultural machinery power/ crop sown area	kW/ha	-	0.006	0.072	0.039
	Agricultural electricity intensity	Rural electricity consumption/gross output value of agriculture, forestry, animal husbandry and fishery*100%	kWh / 10000 yuan	-	0.003	0.089	0.046
	Effective irrigation rate of cultivated land	Effective irrigated area/cultivated area*100%	%	+	0.060	0.040	0.050
	The proportion of financial input in agriculture	Agriculture, forestry and water affairs expenditure/fiscal expenditure*100%	%	+	0.036	0.044	0.040
Environment Friendly	Pesticide application per unit arable area	Pesticide use/year-end arable land area	Tons/ha	-	0.013	0.046	0.030
	Fertilizer application per unit arable area	Fertilizer application of agricultural fertilizers / year-end arable land area	Tons/ha	-	0.028	0.039	0.034
	Film usage per unit arable area	Agricultural plastic film use/year-end arable land area	Tons/ha	-	0.010	0.055	0.033
	Fuel consumption per unit of farm machinery	Agricultural diesel use/total power of agricultural machinery	Tons/kW	-	0.010	0.052	0.031
Ecology Conservation	The proportion of soil erosion control area	Soil erosion control area//area of national territory*100%	%	+	0.110	0.052	0.081
	Forest coverage	Forest area//area of national territory*100%	%	+	0.079	0.034	0.056
	Disaster rate	Damaged area / affected area*100%	%	-	0.020	0.055	0.037
	The proportion of nature reserves	Nature reserve area/area of national territory*100%	%	+	0.107	0.042	0.075
	The proportion of flood removal area	Flood removal area/area of national territory*100%	%	+	0.251	0.050	0.150
Economic Benefits	Grain unit yield	Total grain production/grain sown area	Tons/ha	+	0.045	0.047	0.046
	Agricultural land output rate	Total agricultural output/Sown area of major crops	Yuan/ha	+	0.092	0.057	0.074
	Agricultural labor productivity	Total output value of agriculture, forestry, animal husbandry and fishery / total rural population	Yuan/person	+	0.086	0.055	0.070
	The growth rate of farmers’ net income	(Current net income of rural residents—Previous net income of rural residents)/Previous net income of rural residents*100%	%	+	0.018	0.057	0.038

(2) Independent variable. In this paper, the level of development of agricultural socialized services is measured by the output value of the agricultural, forestry, animal husbandry, and fishery service industry. This industry encompasses various support service activities for agricultural, forestry, animal husbandry, and fishery production activities. Although it does not include miscellaneous scientific, technological, and professional technical service activities, it is similar in concept and content to the agricultural production service industry. It can characterize the level of development of agricultural socialized services [44]. In order to facilitate vertical data comparison, the output value of the agricultural, forestry, animal husbandry, and fishery service industry was standardized to a comparable variable based on 2003. Since 2003, the output value of agriculture, forestry, animal husbandry, and fishery services has been equal to the total output value of agriculture, forestry, animal husbandry, and fishery minus the sum of the four output values of agriculture, forestry, animal husbandry, and fishery.

(3) Moderating variables. Land transfer (*LTR*) is measured by calculating the proportion of the total area of family-contracted land transfer to the area of family-contracted cultivated land. Agricultural technology innovation (*ATI*), measured by the number of patents granted to agricultural enterprises, which includes the sum of invention, utility, and appearance patents granted. Rural human capital (*RHC*), measured by the educational attainment of the population aged six and above [67].

(4) Control variables.To enhance the external validity of the research findings, this paper selects specific control variables that have been shown to have a significant impact on AGD. Specifically, for example, rural population aging (*Aging*) is measured by the proportion of the population aged 65 and over in rural areas. Urbanization rate (*UB*) is measured by the proportion of the urban population in the total population (both agricultural and non-agricultural residents, in the area. Agricultural planting structure (*APS*) is measured by the ratio of the area sown to food crops to the area sown to non-food crops (area sown to crops minus area sown to food crops). Degree of the agricultural scale (*ASD*) is measured by the ratio of the total area sown to the rural population. Industrial structure (*IST*) is measured by the ratio of the value added of the secondary industry to regional GDP.

### Data description and descriptive statistical analysis

We use annual data from 31 provinces (municipalities and autonomous regions in China from 2003 to 2020. The original data for calculating the level of AGD, urbanization rate, disaster rate, agricultural planting structure, degree of agricultural scale, industrial structure, and the output value of the agricultural production service industry were primarily sourced from the Statistical Yearbook of each province (municipalities and autonomous regions), China Environmental Statistical Yearbook, and China Tertiary Industry Statistical Yearbook. The data on authorized patents of agricultural enterprises were obtained from the China Academy for Rural Development-Qiyan China Agri-research Database (CCAD) at Zhejiang University. The data on land transfer were obtained from the China Rural Statistical Yearbook, National Statistics on Rural Economic Situation, and China Rural Management Statistical Annual Report for the respective years. The original data used to calculate calculations on rural population aging and rural population education levels, were sourced from the China Population and Employment Statistical Yearbook. To reduce the volatility of the data and overcome problems such as heteroskedasticity, logarithms were applied to agricultural socialized services, agricultural technology innovation, and rural human capital. Some missing data were imputed using the mean method. The results of descriptive statistical analysis of the main variables are presented in Table 2.

**Table 2:** Descriptive statistical analysis of variables.

Variable classification	Variables	Mean	SD	Min	Max	Obs
Dependent variable	Agricultural green development (*AGD*)	0.31	0.06	0.19	0.55	558
Independent variable	Agricultural socialized services (*ASSV*)	4.26	1.46	0.19	7.11	558
Moderating variables	Land Transfer (*LTR*)	0.21	0.18	0.00	0.91	558
	Agricultural technology innovation (*ATI*)	5.93	1.82	0.00	9.13	558
	Rural Human Capital (*RHC*)	2.09	0.19	1.18	2.54	558
Control variables	Rural population aging (*Aging*)	0.11	0.04	0.05	0.26	558
	Urbanization rate (*UB*)	0.53	0.15	0.20	0.90	558
	Agricultural planting structure (*APS*)	2.90	3.32	0.62	23.08	558
	Degree of agricultural scale-up (*ASD*)	0.26	0.19	0.32	1.37	558
	Industry Structure (*IST*)	0.42	0.08	0.16	0.62	558

### Model setting

#### Benchmark regression model

In existing studies, the bidirectional fixed-effect model is widely used in panel data modeling. This model can consider the differences of independent variables at both the time level and the individual level. Additionally, it helps to avoid missing variable bias and endogeneity problems to a certain extent. Therefore, we establish a bidirectional fixed-effect model to evaluate the causal relationship between agricultural socialized services and AGD. The model is constructed as follows:

$$AGD_{it}=\alpha_{0}+\alpha_{1}\ln ASSV_{it}+{\sum \alpha_{j}Control}_{it}+\mu_{i}+\tau_{t}+\varepsilon_{it}$$ (1)

Herein, the explanatory variable *AGD*_*it*_ is the level of AGD in each province. The core explanatory variable ln*ASSV*_*it*_ is the level of development of agricultural socialized services in each province, *Control*_*it*_ includes control variables such as rural population aging, urbanization rate, planting structure, agricultural scale and industrial structure, *μ*_*i*_ is individual effects, *τ*_*t*_ is time effects, and *ε*_*it*_ is random disturbance terms.

#### Moderating effect model

To examine the moderating effects of land transfer, agricultural technology innovation, and rural human capital on the relationship between agricultural socialized services and AGD, a moderating effect model is constructed based on the benchmark regression model.


$$AGD_{it}=\beta_{0}+\beta_{1}\ln ASSV_{it}+\beta_{2}Mod_{it}+\beta_{3}\ln ASSV_{it}•Mod_{it}+\sum \beta_{j}Control_{it}+\mu_{i}+\tau_{t}+\varepsilon_{it}$$ (2)

Where *Mod*_*it*_ is the moderating variable, including land transfer (*LTR*), agricultural technology innovation (ln*ATI*), and rural human capital (ln*RHC*). In order to address the issue of multicollinearity, the levels of agricultural socialized services, land transfer, agricultural technology innovation, and rural human capital are standardized separately. The interaction term is generated by multiplying each moderating variable by agricultural socialized services. The remaining variables are the same as those in the Eq (1).

#### Spatial Durbin model

In the spatial Durbin model, the influence of geographical location on the relationship between variables is fully considered, especially in the context of spatial lag effect. This model can not only capture the direct spatial interaction of variables but also reveal the indirect effects caused by geographical proximity. By introducing the spatial weight matrix, the spatial Durbin model can more accurately describe the spatial dependence between the observed values, providing a deeper and more comprehensive understanding of spatial phenomena. Therefore, in order to explain the spatial relationship between agricultural socialized services and AGD, this paper adopts the spatial Durbin model to estimate causality. After conducting LM, Robust LM, LR, Wald, and Hausman tests, we selected the bidirectional fixed spatial Durbin model to empirically examine the spatial spillover effect of agricultural socialized services on AGD.


$$\begin{array}{l} AGD_{it}=\alpha +\rho W_{ij}AGD_{it}+\lambda_{1}\ln ASSV_{it}+\lambda_{2}W_{ij}\ln ASSV_{it}+\alpha_{j}\sum Control_{it}\\ +\lambda_{j}\sum W_{ij}Control_{it}+\mu_{i}+\tau_{t}+\varepsilon_{it} \end{array}$$ (3)

Herein, *W*_*ij*_ is the spatial weight matrix, and this paper adopts the spatial geographic distance weight matrix (*W*_1_) for the benchmark analysis and the spatial economic geographic distance weight matrix (*W*_2_) for the robustness analysis. The geographic distance is represented by the spherical distance between two provincial capitals, and its inverse is taken to construct the spatial geographic distance weight matrix (the geographic distance of the same province is 0); the spatial economic geographic distance is represented by the product of the ratio of per capita GDP between two provinces and the spatial geographic distance, and its product is taken to construct the spatial economic distance weight matrix (the spatial economic geographic distance of the same province is 0). Before using the spatial panel econometrics model, it is necessary to use the Moran index to measure the spatial autocorrelation.


$$Moran'sI=\frac{\sum_{i=1}^{n}\sum_{j=1}^{n}W_{ij}(x_{i}-\bar{x})(x_{j}-\bar{x})}{\sum_{i=1}^{n}\sum_{j=1}^{n}W_{ij}(x_{i}-\bar{x})}$$ (4)

Where, *x*_*i*_ denotes the observed value of the spatial cell *i*, *x*_*j*_ denotes the observed value of the spatial cell *j*, $\bar{x}$ is the average value, *n* is the number of spatial cells, *W*_*ij*_ is the spatial weight matrix, the weights *W*_1_ and *W*_2_ are used for the measurement.

## Discussion of results

### Benchmark regression analysis

Before conducting the benchmark regression analysis, the variance inflation factor (VIF) test was used to check the model for multicollinearity. The results indicated that the VIF for each variable was less than 5, with a mean VIF value of 1.72, suggesting no multicollinearity among the variables. Meanwhile, unit root tests were conducted for each variable using the LLC test, Breitung test, IPS test, and Fisher test. It was found that all variables passed at least three of these tests, indicating that the data were stable. The robust standard error was used for estimation in the following regression models.

Table 3 presents the results of the benchmark regression analysis. Based on the regression results of model (1), agricultural socialized services have a positive impact on AGD, even without the inclusion of control variables and time effects. Specifically, a 1% increase in the output value of agricultural socialized services increases the level of AGD by 0.037. When control variables are included in model (2), the results still suggest that agricultural socialized services positively contribute to AGD. Furthermore, agricultural socialized services continue to make an important contribution to AGD after accounting for control variables, time effects, and individual effects in model (3). Overall, the benchmark regression analysis in Table 3 clearly demonstrates the significant promotional effect of agricultural socialized services on the AGD, thus confirming H1a. This conclusion is consistent with those of Yang et al. [18], Qing et al. [22], and Zhang et al. [37]. At present, the development of agricultural socialized services in China is progressing rapidly, playing a positive role in promoting environmentally friendly agricultural production. For instance, professional technical services such as soil testing, customized fertilization, and integrated disease and pest management offered by socialized service organizations effectively facilitate the decrease in pesticide and fertilizer usage and the recycling of straw into the fields [26,27,68]. Consequently, this promotes the enhancement and sustainable development of the agricultural ecological environment. With the continuous development of information and intelligent technology, an increasing number of social service organizations are starting to utilize big data, artificial intelligence, and other advanced technologies in agricultural production. This trend is enhancing the accuracy and efficiency of agricultural green production [69–71]. Therefore, with the expansion of the scale of agricultural socialized services, the level of AGD also shows an increasing trend.

**Table 3:** Results of benchmark regression.

Variables	(1)	(2)	(3)
	*AGD*	*AGD*	*AGD*
ln*ASSV*	0.037***	0.016***	0.010***
	(22.43)	(7.53)	(5.09)
*Control variables*	No	Yes	Yes
*μ*_*i*_	Yes	Yes	Yes
*τ*_*t*_	No	No	Yes
*N*	558	558	558
*Adj*. *R*^*2*^	0.904	0.935	0.944

Notes: t statistics are reported in parentheses

* p<0.1

** p<0.05

*** p<0.01.

### Robustness test

In the empirical analysis, errors in method and variable selection may lead to biased results. To ensure the robustness of the results, this paper employs the method of replacing the core explanatory variables, reducing the sample size, and eliminating outliers for validation. The specific empirical results are as follows.

#### Replacing the dependent variable

Given that the entropy method fails to account for the correlation between indicators, it may introduce biases in assessing the level of green development in agriculture, ultimately leading to biased regression results. To rectify this issue, we employ the CRITIC model to recalculate the weights and redetermines the combined weights (namely, W_con=0.5*W_en+0.5*W_cri) by incorporating the weights derived from the entropy weight method. This approach allows us to obtain a more comprehensive representation of the indicator data’s characteristics and the relationships among them. This approach ensures a more accurate measurement of the level of agricultural green development (AGD). The results presented in models (4) and (5) of Table 4 demonstrate that agricultural socialized services significantly contribute to promoting AGD, which is consistent with the findings outlined in Table 3.

**Table 4:** Results of robustness tests.

Variables	(4)	(5)	(6)	(7)
	*AGD*	*AGD*	*AGD*	*AGD*
ln*ASSV*	0.008***	0.007***	0.006***	0.007***
	(4.18)	(3.63)	(3.54)	(3.75)
*Control Variables*	No	Yes	Yes	Yes
*μ*_*i*_	Yes	Yes	Yes	Yes
*τ*_*t*_	Yes	Yes	Yes	Yes
*N*	558	558	403	486
*Adj*. *R*^*2*^	0.884	0.889	0.960	0.939

Notes: t statistics are reported in parentheses

* p<0.1

** p<0.05

*** p<0.01.

#### Selecting sub-samples

In October 2015, the Fifth Plenary Session of the 18th Central Committee of China proposed a new development concept for the first time. This concept is based on the principles of "innovation, coordination, green, openness, and sharing." This concept placed significant emphasis on the importance of "green" development, which has had a profound impact on the development of agriculture. This concept has dramatically stimulated agriculture and rural areas to follow the path of AGD unwaveringly. To eliminate the influence of this policy, the sample size is reduced to 2015 to confirm if the positive relationship between agricultural socialized services and AGD remains robust. Model (6) in Table 4 presents the test results from 2003–2015 and indicates that the promotion of agricultural socialized services has a significant impact on AGD is consistently aligned with the benchmark regression results.

#### Removing specific samples

Considering the advantages of municipalities directly under the central government in terms of political and economic development, four municipalities, namely Beijing, Shanghai, Tianjin, and Chongqing, are excluded from the total samples for robustness testing. Model (7) in Table 4 demonstrates that the coefficient representing the impact of agricultural socialized services on AGD remains significantly positive even after excluding the samples from the four municipalities directly under the central government. This suggests that the conclusion regarding the impact of agricultural socialized services on AGD is robust.

### Endogeneity test

Considering the potential endogeneity of reciprocal causality in the model, highway mileage was chosen as the instrumental variable. Specifically, the increase in highway mileage will enhance transport accessibility, thereby promoting the provision and utilization of agricultural socialized services. At the same time, highway mileage is a relatively objective and observable index that is not easily affected by agricultural socialized services. Therefore, it can be used as an independent variable for endogeneity testing. In the endogeneity test, the two-stage least squares method (IV - 2SLS) and the system generalized method of moments (IV—GMM) model were used to estimate the impact of agricultural socialized services on AGD. According to the estimated results in Table 4, the F-value obtained in one stage is 459.09, significantly exceeding the critical value of 10. This suggests a strong correlation between the instrumental variable and the endogenous variable. The Cragg-Donald Wald F statistics for models (9) and (10) were 150.714, significantly exceeding the 10% critical empirical value of 16.38. Additionally, the Kleibergen-Paap rk LM statistic rejects the non-discernible null hypothesis at the 1% significance level. In addition, the Hansen J statistic shows no problem of overidentification, indicating that the selected instrumental variables are appropriate. Comparing model (3) in Table 3 with models (9) and (10) in Table 5, agricultural socialized services still significantly promote AGD after including highway mileage, which is consistent with the benchmark regression results.

**Table 5:** Results of endogeneity test.

Variables	(8)	(9)	(10)
	*AGD*	*AGD*	*AGD*
*Highway*	0.135***		
	(21.43)		
ln*ASSV*		0.006***	0.006***
		(3.03)	(3.03)
*Kleibergen-Paap rk LM statistic*		150.714 < 0.00>	150.714 < 0.00>
*Cragg-Donald Wald F statistic*		511.871 <16.38>	511.871 <16.38>
*Hansen J statistic*		0.000	0.000
*Control Variables*	Yes	Yes	Yes
*μ*_*i*_	Yes	Yes	Yes
*τ*_*t*_	Yes	Yes	Yes
*N*	527	527	527
*Adj*. *R*^*2*^		0.569	0.569
*F*	459.09	108.01	114.23

Notes: t statistics are reported in parentheses

* p<0.1

** p<0.05

*** p<0.01.

#### Heterogeneity test

Given the main issue of the aging population in China’s rural areas, varying degrees of aging may have diverse effects on the adoption of agricultural socialized services and AGD. Therefore, it is divided into a region of low aging and a region of high aging based on the mean value for conducting a heterogeneity test.

Table 6 presents the results of the heterogeneity test and the test for differences in coefficients between groups. The significant difference in coefficients between groups indicate that the coefficients are comparable, suggesting the presence of heterogeneity. It shows that agricultural socialized services play a significant role in promoting AGD in the low-aging region, while they are not significant in the high-aging region. It may be because the overall education level in the high-aging region is low, and the acceptance of advanced knowledge, technology, and service modes is not high. Furthermore, farmers in this region prefer to conduct decentralized and small-scale operations. As a result, the positive impact of agricultural socialized services cannot be realized.

**Table 6:** Results of heterogeneity test.

Variables	Low Aging Region	High Aging Region
	*AGD*	*AGD*
ln*ASSV*	0.008***	-0.010
	(4.35)	(-1.41)
*Control Variables*	Yes	Yes
*μ*_*i*_	Yes	Yes
*τ*_*t*_	Yes	Yes
*N*	319	239
*Adj*. *R*^*2*^	0.953	0.946
Observed difference	0.018	
Empirical p-value	0.000***	

Notes: In the test for heterogeneity in the Low Aging Region and High Aging Region, aging was excluded as a control variable. Empirical p-value is used to test the significance of the coefficient difference between groups, obtained by bootstrapping 1000 times. t statistics are reported in parentheses

* p<0.1

** p<0.05

*** p<0.01.

### Spatial spillover effect

**Fig 2:**
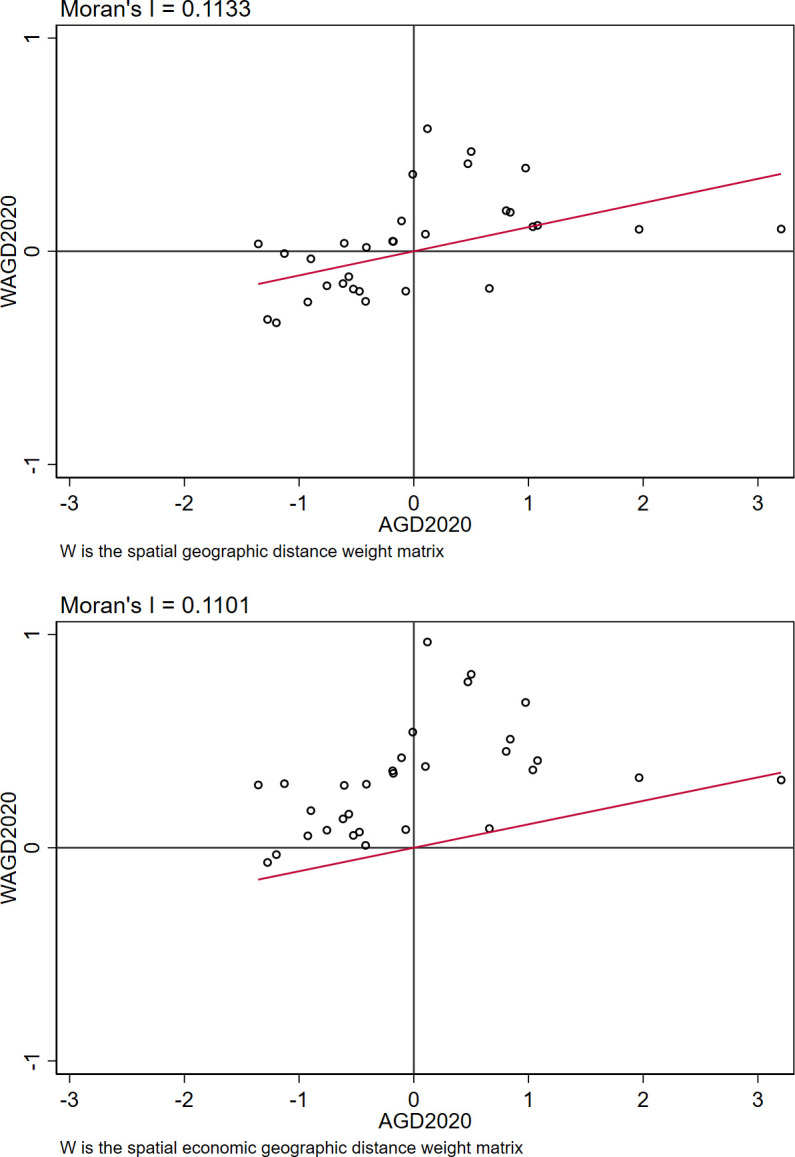
Moran index scatter plot for AGD (2020).

The results presented in Table 7 demonstrate that the Moran’s I index for the period of 2003–2020 exhibits significant positive values when employing spatial weights *W*_1_ and *W*_2_, respectively. This indicates a positive spatial clustering relationship within China’s AGD, with each province displaying robust spatial correlation. Furthermore, as illustrated in Fig 2, a predominant trend of positive spatial clustering of AGD is observed across the majority of provinces in 2020. Combined with the above results, it is appropriate to analyze the spatial effects of AGD in China using a spatial econometric model. Therefore, the model in Eq (3) is utilized to estimate the spatial spillover effect of agricultural socialized services on AGD.

**Table 7:** Moran’s I for AGD.

Year	*W*_1_	*W*_2_	Year	*W*_1_	*W*_2_
2003	0.148***	0.162***	2012	0.197***	0.207***
	(5.41)	(5.26)		(6.61)	(6.30)
2004	0.163***	0.176***	2013	0.200***	0.211***
	(5.78)	(5.59)		(6.68)	(6.37)
2005	0.151***	0.166***	2014	0.198***	0.211***
	(5.46)	(5.35)		(6.64)	(6.39)
2006	0.156***	0.167***	2015	0.176***	0.181***
	(5.57)	(5.37)		(6.01)	(5.63)
2007	0.154***	0.167***	2016	0.18***	0.187***
	(5.51)	(5.35)		(6.13)	(5.77)
2008	0.159***	0.17***	2017	0.155***	0.162***
	(5.61)	(5.38)		(5.53)	(5.19)
2009	0.171***	0.187***	2018	0.137***	0.142***
	(5.90)	(5.79)		(5.09)	(4.73)
2010	0.181***	0.193***	2019	0.126***	0.132***
	(6.21)	(5.95)		(6.74)	(4.46)
2011	0.189***	0.199***	2020	0.113***	0.110***
	(6.40)	(6.09)		(4.39)	(3.88)

Notes:z statistics are reported in parentheses

* p<0.1

** p<0.05

*** p<0.01.

Table 8 delineates the spatial spillover effects at spatial weights of *W*_1_ and *W*_2_ in both model (11) and model (12). Upon inspecting the coefficients of the interaction terms of spatial weights *W*_1_ and *W*_2_ and agricultural socialized services in Table 8, it becomes evident that they are significantly positive. This indicates that agricultural socialized services have a significant positive spatial influence on AGD. Further analysis reveals that the direct and total effects of agricultural socialized services on AGD are statistically significant at the 10% level. However, the coefficients representing the indirect effects do not reach significance. This implies that the spatial spillover effect of agricultural socialized services are mainly manifested through direct channels. In simpler terms, the development of agricultural socialized services primarily promotes AGD within the local region, with limited evidence of effects on neighboring regions, rendering H1b inconclusive. This phenomenon can be attributed to the fact that the development of agricultural socialized services is still in its infancy. The scale of the current flow of these services across regions is relatively small, which may hinder their broader impact. Consequently, this limitation restricts their potential to significantly increase AGD on a larger scale.

**Table 8:** Test results of spatial spillover effect.

Variables	(11)	(12)
	*AGD*	*AGD*
ln*ASSV*	0.009***	0.009***
	(5.13)	(4.94)
*W*×ln*ASSV*	0.025***	0.022**
	(2.29)	(2.07)
*LR_Direct*	0.009***	0.009***
	(4.51)	(4.40)
*LR_Indirect*	0.010	0.009
	(1.55)	(1.37)
*LR_Total*	0.019***	0.018***
	(3.13)	(2.85)
*rho*	-0.790***	-0.912***
	(-4.58)	(-4.83)
*sigma2_e*	0.000***	0.000***
	(16.49)	(16.33)
*Control Variables*	Yes	Yes
*μ*_*i*_	Yes	Yes
*τ*_*t*_	Yes	Yes
*N*	558	558
*R* ^*2*^	0.178	0.236

Notes: z-statistics are reported in parentheses

* p<0.1

** p<0.05

*** p<0.01.

### Moderating effects

Building on the preceding theoretical analysis, it is evident that land transfer, agricultural technology innovation, and rural human capital exert moderating influences on the relationship between agricultural socialized services and AGD. This implies that the influence of agricultural socialized services on AGD varies depending on the levels of land transfer, agricultural technology innovation, and rural human capital. Table 9 presents a detailed account of the individual moderating effects of land transfer, agricultural technology innovation, and rural human capital, shedding light on their nuanced impact.

**Table 9:** Moderating effects of land transfer, agricultural technology innovation, and rural human capital.

Variables	(13)	(14)	(15)
	*AGD*	*AGD*	*AGD*
ln*ASSV*	0.010***	0.010***	0.010***
	(5.32)	(4.80)	(5.33)
*LTR*	-0.020		
	(-1.46)		
ln*ASSV*×*LTR*	0.015***		
	(3.07)		
ln*ATI*		0.003**	
		(2.22)	
ln*ASSV*×ln*ATI*		0.001***	
		(4.29)	
ln*RHC*			0.049**
			(2.53)
ln*ASSV*×ln*RHC*			0.016***
			(4.56)
*Control Variables*	Yes	Yes	Yes
*μ*_*i*_	Yes	Yes	Yes
*τ*_*t*_	Yes	Yes	Yes
*N*	558	558	558
*Adj*. *R*^*2*^	0.945	0.946	0.946

Notes: t statistics are reported in parentheses

* p<0.1

** p<0.05

*** p<0.01.

1. Land transfer has a positive moderating effect. The coefficient of the cross-product term between land transfer and agricultural socialized services is positive and significant at the 1% level, which is consistent with the main effect. The marginal contribution of agricultural socialized services to AGD is a function of land transfer, and its marginal contribution is 0.01+0.015*LTR*. It indicates that the higher the rate of land transfer, the greater the promotional effect of agricultural socialized services on AGD, thus confirming H2. This could be attributed to the current trend of rising land transfers. As the rate of land transfer increases, there is a growing recognition of moderate-scale land management. This, in turn, provides a low-cost platform for the expansion of socialized agricultural services. The availability of such low-cost land scales encourages greater participation of agricultural socialized services in various agricultural activities. This, in turn, creates favorable conditions for the implementation of environmentally friendly and efficient agricultural production practices.
2. Agricultural technology innovation has a positive moderating effect. The coefficient of the cross-product term between agricultural technology innovation and agricultural socialized services is positive and significant at the 1% level, which is consistent with the main effect. The marginal contribution of agricultural socialized services to AGD is a function of agricultural technological innovation,and its marginal contribution is 0.01+0.001ln*ATI*. It indicates that the higher the level of agricultural technology innovation, the greater the promotional effect of agricultural socialized services on AGD, thus verifying H3. This may be because with the continuous progress of agricultural technological innovation, the impact of agricultural socialized services becomes increasingly significant. They can not only alleviate problems arising from the aging of the rural labor force, such as the issue of non-planting, but also facilitate large-scale production. Furthermore, the introduction of these services can lead to a reduction in the use of chemical inputs such as fertilizers and pesticides. This practice is not only aligned with environmentally friendly practices but also contributes to the enhancement of green agricultural total factor productivity. Ultimately, this comprehensive approach promotes sustainable agricultural development, in line with the broader objectives of fostering environmental awareness and implementing efficient agricultural practices.
3. Rural human capital has a positive moderating effect. The coefficient of the cross-product term between rural human capital and agricultural socialized services is positive and significant at the 1% level, which is consistent with the main effect. The marginal contribution of agricultural socialized services to AGD is a function of rural human capital, namely, 0.01+0.016ln*RHC*. This result indicates that as the educational level of the rural labor force improves, the positive impact of agricultural socialized services on AGD becomes more evident, thus confirming H4. This trend can be attributed to the increasing environmental awareness of farmers as they become more educated. As the level of education improves, farmers gain a deeper understanding of the importance of agricultural socialized services and realize that the sustainable development of agriculture is closely linked to their own economic well-being. As a result, farmers are more willing to adopt innovative farming methods and utilize agricultural socialized services to enhance agricultural productivity.

## Conclusion, policy implications and limitations

### Conclusion

Unlike other scholars who have explored the impact of agricultural socialized services on farmers’ adoption of green production technologies from a micro perspective (e.g., Liu et al., 2022; Cheng, 2022; Zhang et al., 2022), we utilized data from 31 provinces in China spanning from 2003 to 2020. We estimated the influence of agricultural socialized services on AGD and its spatial effects at the macro level by employing the bidirectional fixed-effect model and the spatial Durbin model. In addition, we employed the moderating effects model to examine the moderating influences of land transfer, agricultural technological innovation, and rural human capital on the impact of agricultural socialized services on agricultural green development. Our findings suggest that agricultural socialized services significantly promote AGD, which is consistent with the studies by Yang et al. (2022), Qing et al. (2022), and Zhang et al. (2023). Through further analysis of heterogeneity, we observe that the positive impact of agricultural socialized services on AGD is more pronounced in regions characterized by lower levels of aging. By integrating spatial and moderating effect analyses, we ascertain that the promotion effect of agricultural socialized services on AGD does not exhibit significant spatial spillover effect. The presence of land transfer, agricultural technological innovation, and rural human capital exert a positive moderating influence on the role of agricultural socialized services in promoting AGD.

### Policy implications

This paper provides several insights to accelerate AGD. Firstly, the government should increase financial support for the construction of agricultural socialized service organizations. It should actively encourage agricultural service enterprises, farmers’ cooperatives, rural collective economic organizations, grassroots supply and marketing cooperatives, and other entities to participate in building the agricultural socialized service system. The government should vigorously support all types of agricultural socialized service providers to promote green production, and enhance the level of marketization in agricultural services and boost research and development capabilities in agricultural green production technologies, so as to maximize the spillover effects of agricultural socialized service technologies. Secondly, the government should promote the transfer of rural farmland, enhance relevant laws, regulations, and policies, and protect the land rights and interests of farmers. Meanwhile, a service platform should be established for the transfer of farmland. This platform should offer services such as releasing information on farmland transfers, facilitating transaction matches, encouraging the involvement of agricultural enterprises, farmers’ professional cooperatives, and other key stakeholders, and promoting the adoption of appropriate scales of farmland operation. These efforts aim to create favorable conditions for the advancement of green agriculture. Additionally, the government should increase investment in rural education, improve educational facilities and teachers’ salaries, establish agricultural science and technology courses, and train professional personnel. Technical training and professional guidance must also be provided to empower farmers with the knowledge and skills necessary to adopt and apply green agricultural technologies, thereby offering talent support for the advancement of green agriculture. Finally, the government should also utilize a variety of channels, such as the media and the Internet, to publicize the importance of green agriculture and to enhance public awareness and acceptance of green agriculture. At the same time, training and demonstration activities should be organized to enable farmers to experience firsthand the benefits of green production methods. In addition, a certification and reward system for green agricultural products should be established to incentivize farmers to embrace green production methods actively, thereby enabling them to produce high-quality green agricultural products to meet market demands and foster sustainable agricultural development.

### Limitations and future research directions

However, there are some limitations to this study that need to be highlighted. First, since this paper relies on macro data, there may be a delay in the external disclosure of many data points, or even a significant amount of missing data, which could potentially limit the scope and depth of the analyses in this study. Therefore, in future research, we can explore methods to obtain more comprehensive and accurate data, such as conducting surveys or interviews, which could enhance the study’s appeal. Secondly, measuring the output value of agricultural socialized services remains a challenge. Currently, there are no specific statistics on agricultural socialized services, which can only be characterized by proxy variables. This may affect the estimation results of this paper. In future research, we can develop a set of indicators that comprehensively reflect the output value and benefits of agricultural socialized services by thoroughly analyzing and integrating existing data, and by considering the characteristics and principles of agricultural socialized services. Finally, although we have attempted to analyze the regional variations in the influence of agricultural socialized services on AGD, we have not considered the more specific geo-environmental factors, such as differences in climate, topography, soil, and other natural conditions. Therefore, in future research, we can further incorporate geographical information technology. By utilizing spatial analysis and econometric modeling, we can delve deeper into the impacts of agricultural socialized services on AGD under various geographical conditions.

## Supporting information

S1 Data (ZIP)

## References

[1] Chen C, Park T, Wang X, Piao S, Xu B, Chaturvedi R. et al. China and India lead in greening of the world through land-use management. Nature Sustainability, 2019; 2: 122–129. doi: 10.1038/s41893-019-0220-7 30778399 PMC6376198

[2] Sun X, Xiang P, Cong K. Research on early warning and control measures for arable land resource security. Land Use Policy. 2023; 128: 106601. 10.1016/j.landusepol.2023.106601.

[3] Ma W, Li J, Ma L, Wang F, Sisák I, Cushman G, et al. Nitrogen flow and use efficiency in production and utilization of wheat, rice, and maize in China. Agricultural Systems, 2008; 99 (1): 53–63.

[4] Huang J, Huang Z, Jia X, Hu R, Xiang C. Long-term reduction of nitrogen fertilizer use through knowledge training in rice production in China.Agricultural Systems, 2015;135: 105–111. 10.1016/j.agsy.2015.01.004.

[5] Chen Z, Sarkar A, Rahman A, Li X, Xia X. Exploring the drivers of green agricultural development (GAD) in China: A spatial association network structure approaches. Land Use Policy, 2022; 112: 105827. 10.1016/j.landusepol.2021.105827.

[6] Ren S, Du M, Bu W, Lin T. Assessing the impact of economic growth target constraints on environmental pollution: Does environmental decentralization matter? Journal of Environmental Management, 2023; 336: 117618. doi: 10.1016/j.jenvman.2023.117618 36905691

[7] Keshavarz M, Sharafi H. Scaling up climate-smart regenerative agriculture for the restoration of degraded agroecosystems in developing countries. Sustainable Production and Consumption, 2023; 38: 159–173. 10.1016/j.spc.2023.04.003.

[8] Song G, Ren G. Spatial response of cultivated land use efficiency to the maize structural adjustment policy in the “sickle bend” region of China: An empirical study from the cold area of Northeast. Land Use Policy, 2022; 123: 106421, https://dol.org/10.1016/j.landusepol.2022.106421.

[9] Lu L, Cheng H, Pu X, Wang J, Cheng Q, Liu X. Identifying organic matter sources using isotopic ratios in a watershed impacted by intensive agricultural activities in Northeast China. Agriculture, Ecosystems & Environment, 2016; 222: 48–59. 10.1016/j.agee.2015.12.033.

[10] Zhang H, Zhang J, Song J. Analysis of the threshold effect of agricultural industrial agglomeration and industrial structure upgrading on sustainable agricultural development in China. Journal of Cleaner Production, 2022; 341: 130818. 10.1016/j.jclepro.2022.130818.

[11] Guo H, Liu X. Time-space evolution of China’s agricultural green total factor productivity. Chinese Journal of Management Science, 2020; 8(09): 66–75. doi: 10.16381/j.cnki.issn1003-207x.2020.09.007

[12] Shen X, Duan J, Zhu S. Exploring research on socialized services patterns for green agricultural production. Chinese Journal of Agricultural Resources and Regional Planning, 2020; 41(01): 15–20.

[13] Wei H, Yuan P, Lu Q. Historical evolution and theoretical innovation of agricultural and rural development research in China, Reform, 2020; (10): 5–18.

[14] Lu Q. China’s agricultural productive service industry: Review of 70 years of development,evolutionary logic and future prospects, Economist, 2019; (11): 5–13. doi: 10.16158/j.cnki.51-1312/f.2019.11.002

[15] Ji M. Agricultural Productive Service Industry: The third momentum in Chinese agricultural modernization history. Issues in Agricultural Economy, 2018; (03): 9–15. doi: 10.13246/j.cnki.iae.2018.03.003

[16] Huan M, Li Y, Chi L, Zhan S. The effects of agricultural socialized services on sustainable agricultural practice adoption among smallholder farmers in China. Agronomy, 2022; 12: 2198–2198.

[17] Zhu Y, Deng J, Wang M, Tan Y, Yao W, Zhang Y. Can agricultural productive services promote agricultural environmental efficiency in China? International Journal of Environmental Research and Public Health. 2022; 19(15): 9339. doi: 10.3390/ijerph19159339 35954694 PMC9368607

[18] Yang C, Zeng H, Zhang Y. Are socialized services of agricultural green production conducive to the reduction in fertilizer input? Empirical evidence from rural China. International Journal of Environmental Research and Public Health. 2022; 19(22): 14856. doi: 10.3390/ijerph192214856 36429575 PMC9690766

[19] Dong H, Guo X. Productive services and traditional agriculture: reform or continuation: an empirical analysis based on 501 family questionnaires of farmers in Sichuan province. Economist, 2014; 6: 84–90. doi: 10.16158/j.cnki.51-1312/f.2014.06.006

[20] Cheng C, Gao Q, Qiu Y. Assessing the ability of agricultural socialized services to promote the protection of cultivated land among farmers. Land. 2022; 11(8): 1338. doi: 10.3390/land11081338

[21] Sun X, Liu Y. Can land trusteeship promote farmers’ green production? Chin. Rural Econ, 2019; 10:60–80.

[22] Qing C, Zhou W, Song J, Deng X, Xu D. Impact of outsourced machinery services on farmers’ green production behavior: Evidence from Chinese rice farmers. Journal of Environmental Management, 2023; 327: 116843. doi: 10.1016/j.jenvman.2022.116843 36459784

[23] Lu H, Chen Y, Hu H, Gen X. Can agricultural socialized services promote farmers to adopt pro-environment agricultural technologies? Journal of Agrotechnical Economics, 2021; 3: 36–49. doi: 10.13246/j.cnki.jae.2021.03.003

[24] Lewis B D, Pattinasarany D. Determining citizen satisfaction with local public education in indonesia: the significance of actual service quality and governance conditions. Growth & Change, 2010; 40(1): 85–115.

[25] Gideon D A. Do agricultural extension services promote adoption of soil and water conservation practices? Evidence from Northern Ghana. Journal of Agriculture and Food Research, 2022; 10: 100381. 10.1016/j.jafr.2022.100381.

[26] Yan A, Luo A, Luo X, Huang Y. Influence of socialized services on farmers’ pesticide reduction behavior. Journal of Arid Land Resources and Environment, 2021; 35: 91–97.

[27] He W, Liu Y, Sun H, Taghizadeh-Hesary F. How does climate change affect rice yield in China? Agriculture. 2020; 10(10):441. 10.3390/agriculture10100441.

[28] Xu Q, Zhu P, Tang L. Agricultural services: Another way of farmland utilization and its effect on agricultural green total factor productivity in China. Land. 2022; 11(8): 1170. 10.3390/land11081170.

[29] Chen J, Zhang D, Chen Z, Li Z, Cai Z. Effect of agricultural social services on green production of natural rubber: Evidence from hainan. China. Sustainability, 2022; 14(21): 14138. 10.3390/su142114138.

[30] Chen T, Rizwan M, Abbas A. Exploring the role of agricultural services in production efficiency in chinese agriculture: A case of the socialized agricultural service system. Land. 2022; 11(3): 347. 10.3390/land11030347.

[31] Sun D, Lu Y, Tian X. The impact of productive services on the technical efficiency of Chinese rice production—Based on an empirical analysis of data from microscopic surveys in the four provinces of Jiangsu, Zhejiang, Shu, and Guangzhou. Chinese Rural Economy, 2016; (08): 70–81.

[32] Lin Y, Hu R, Zhang C, Chen K. The role of public agricultural extension services in driving fertilizer use in rice production in China. Ecological Economics, 2022; 200:107513. 10.1016/j.ecolecon.2022.107513.

[33] Bambio Y, Agha S. Land tenure security and investment: Does strength of land right really matter in rural Burkina Faso. World Development, 2018; 111: 130–147.

[34] Liu H, Han X, Xue Y, Lv J. The logic of agricultural productive services affecting fertilizer reduction: Sub-stitution and matching. Journal of Arid Land Resources and Environment, 2022; 36(04): 32–38.

[35] Cheng Y, Zhang D, Wang X. Green development effect of agricultural socialized services: An analysis based on farming households’ perspective. Resources Science, 2022; 44(9): 1848–1864. doi: 10.18402/resci.2022.09.09

[36] Zhang L, Yang G, Li H. How to incorporate smallholder farmers into the green development of agriculture: An exploration based on outsourcing services. Journal of Huazhong Agricultural University (Social Sciences Edition), 2022; (04): 53–61. doi: 10.13300/j.cnki.hnwkxb.2022.04.005

[37] Shi Z, Fu Y. Agricultural socialized service organization, land scale and the contradiction between farmers’green production willingness and behavior. Journal of China Agricultural University, 2022; 27(03): 240–254.

[38] Chen X, Liu T. Can agricultural socialized services promote the reduction in chemical fertilizer? analysis based on the moderating effect of farm size? International Journal of Environmental Research and Public Health. 2023; 20(3): 2323. doi: 10.3390/ijerph20032323 36767688 PMC9916101

[39] Zhang M, Tong T, Chen Z. Can socialized service of agricultural production improve agricultural green productivity? South China Journal of Economics, 2023; (01): 135–152. doi: 10.19592/j.cnki.scje.400099

[40] Cao T, Zhou J, Zou W. Effects of land trusteeship on fertilizer input reduction: Mechanism and empirical test. Journal of Arid Land Resources and Environment, 2022; 6(06): 34–40. doi: 10.13448/j.cnki.jalre.2022.144

[41] Ma W, Abdulai A, Goetz R. Agricultural cooperatives and investment in organic soil amendments and chemical fertilizer in China. American Journal of Agricultural Economics, 2018; 100, 502–520.

[42] Li C, Xu J, Wang Y. Can socialized service of agricultural green production improve agricultural green productivity? Journal of Agrotechnical Economics, 2021; (09): 36–49. doi: 10.13246/j.cnki.jae.2021.09.003

[43] Li R, Yu Y. Impacts of green production behaviors on the income effect of rice farmers from the perspective of outsourcing services: Evidence from the rice region in northwest China. Agriculture. 2022; 12(10): 1682. 10.3390/agriculture12101682.

[44] Zhang H, Guo X. The promotion effect of agricultural producer services on agricultural total factor productivity: Regional differences and spatial effect. Journal of Agrotechnical Economics, 2021; (05): 93–107.

[45] Tang W, Zhou F. Spatial effect and influence path of agricultural production-oriented service on farmers’ income increase. Journal of Hunan University of Humanities, Science and Technology, 2022; 39(02): 67–72.

[46] Fang S, Wei L, Wu J. The spatial spillover effect of agricultural mechanization and its distribution pattern: The perspective of interregional-service of agricultural machinery. Journal of Management World, 2017; (11): 65–78+187–188.

[47] Yu S, Liu T, Cao B. Effects of agricultural mechanization service on the cost efficienc of grain product—Evidence from wheat-producing areas in China. Journal of Huazhong Agricultural University (Social Sciences Edition), 2019; (04): 81–89+173. doi: 10.13300/j.cnki.hnwkxb.2019.04.009

[48] Mi Q, Li X, Gao J. How to improve the welfare of smallholders through agricultural production outsourcing: Evidence from cotton farmers in Xinjiang, Northwest China. Journal of Cleaner Production, 2020; 256:120636. 10.1016/j.jclepro.2020.120636.

[49] Zhang L, Luo B. Agricultural Chemical Reduction: The logic and evidence based on farmland operation scale of households. Chinese Rural Economy, 2020; (02): 81–99.

[50] Carter M R, Yao Y. Local versus global separability in agricultural household models: The factor price equalization effect of land transfer rights. American Journal of Agricultural Economics, 2002; 84(3): 702–715.

[51] Gai Q, Cheng M, Zhu X, Shi Q. Can land rent improve land allocations efficiency?—Evidence from national fixed point survey. China Economic Quarterly, 2020; 20(05): 321–340.

[52] Hu X, Chen X, Mi Y. The impact of agricultural land consolidation and titling policies on the development of agricultural scale management: evidence from quasi-natural experiments. Chinese Rural Economy, 2018; (12): 83–102.

[53] Liang Z, Zhang L, Zhang J. Land inward transfer, plot scale and chemical fertilizer reduction: An empirical analysis based on main rice-producing areas in Hubei province. China Rural Survey, 2020; (05): 73–92.

[54] Zhang F, Wang F, Hao R, Wu L. Agricultural science and technology innovation, spatial spillover and agricultural green development—Taking 30 provinces in China as the research object. Applied Sciences. 2022; 12(2): 845. 10.3390/app12020845.

[55] He W, Li E, Cui Z. Evaluation and influence factor of green efficiency of China’s agricultural innovation from the perspective of technical transformation. Chinese Geographical Science, 2021; 31 (2): 313–328.

[56] Eanes F R, Singh A S, Bulla B R, Ranjan P, Fales M, Wickerham B. et al.Crop advisers as conservation intermediaries: Perceptions and policy implications for relying on nontraditional partners to increase U.S. farmers’ adoption of soil and water conservation practices. Land Use Policy, 2019; 81:360–370. 10.1016/j.landusepol.2018.10.054.

[57] Sun Z, Wang L, Li X. Population aging, socialized agricultural services and agricultural high quality development. Journal of Guizhou University of Finance and Economics, 2022; (03): 37–47.

[58] Battese G E, Coelli T J. A model for technical inefficiency effects in a stochastic frontier production for panel data. Empirical Economics, 1995; 20: 325–332. 10.1007/BF01205442.

[59] Zhao K, Zhang R, Sun P. The impacts of capital endowment on farmers’ adoption behaviors of agricultural socialization services: From the perspective of family life cycle. Research of Agricultural Modernization, 2022; 43(1): 121–133. doi: 10.13872/j.1000-0275.2021.0118

[60] Liu Y, Sun D, Wang H, Wang X, Yu G, Zhao X. An evaluation of China’s agricultural green production: 1978–2017. Journal of Cleaner Production, 2020; 243: 118483. 10.1016/j.jclepro.2019.118483.

[61] Liu Z, Zhang M, Li Q, Zhao X. The impact of green trade barriers on agricultural green total factor productivity: Evidence from China and OECD countries. Economic Analysis and Policy, 2023; 78: 319–331. 10.1016/j.eap.2023.03.011.

[62] Fang L, Hu R, Mao H, Chen S. How crop insurance influences agricultural green total factor productivity: Evidence from Chinese farmers. Journal of Cleaner Production, 2021; 321, 128977. 10.1016/j.jclepro.2021.128977.

[63] Song Y, Zhang B, Wang J, Kwek K. The impact of climate change on China’s agricultural green total factor productivity. Technological Forecasting and Social Change, 2022; 185: 122054. 10.1016/j.techfore.2022.122054.

[64] Huang H, Mo R, Chen X. New patterns in China’s regional green development: An interval Malmquist–Luenberger productivity analysis. Structural Change and Economic Dynamics, 2021; 58: 161–173. 10.1016/j.strueco.2021.05.011.

[65] Wei Q, Zhang B, Jin S. A study on construction and regional comparison of agricultural green development index in China. Issues in Agricultural Economy, 2018; 11, 11–20. doi: 10.13246/j.cnki.iae.2018.11.002

[66] Guo H, Xu S. Measurement of the spatial complexity and its influencing factors of agricultural green development in China. Sustainability. 2020; 12(21): 9259. 10.3390/su12219259.

[67] Luo H, Hu Q. A re-examination of the influence of human capital on urban-rural income gap in China: College enrollment expansion, digital economy and spatial spillover,Economic Analysis and Policy, 2021; 81: 494–519. 10.1016/j.eap.2023.12.018.

[68] Zhang X, Yan T. Analysis on the impact of agricultural technical services on farmers’ straw retuming behavior under the background of labor transfer.Taking Hubei Province as an example. Journal of China Agricultural University, 2021; 26(01): 196–207.

[69] Lioutas E D, Charatsari C, La Rocca G, De Rosa M. Key questions on the use of big data in farming: An activity theory approach. NJAS: Wageningen Journal of Life Sciences, 2019; 90–91(1): 1–12. 10.1016/j.njas.2019.04.003.

[70] Benyam A, Soma T, Fraser E. Digital agricultural technologies for food loss and waste prevention and reduction: Global trends, adoption opportunities and barriers. Journal of Cleaner Production, 2021; 323: 129099. 10.1016/j.jclepro.2021.129099.

[71] Shen Z, Wang S, Boussemart J, Hao Y. Digital transition and green growth in Chinese agriculture, Technological Forecasting and Social Change, 2022; 181:121742. 10.1016/j.techfore.2022.121742.

